# Fortification of Durum Wheat Pasta with Mealworm (*Tenebrio molitor*) Powder: Physicochemical, Nutraceutical, and Sensory Effects

**DOI:** 10.3390/molecules31020298

**Published:** 2026-01-14

**Authors:** Ewelina Zielińska, Paulina Sidor, Urszula Pankiewicz

**Affiliations:** 1 Department of Analysis and Food Quality Assessment, University of Life Sciences in Lublin, Skromna Str. 8, 20-704 Lublin, Poland; urszula.pankiewicz@up.edu.pl; 2 Scientific Students Group of Food Analysts, Department of Analysis and Food Quality Assessment, University of Life Sciences in Lublin, Skromna Str. 8, 20-704 Lublin, Poland; sidor102000@gmail.com

**Keywords:** edible insects, protein fortification, antioxidant activity, mineral enrichment, predicted glycemic index, sensory evaluation

## Abstract

Edible insects are gaining popularity as an alternative food source, highlighting the urgent need for research on their incorporation into traditional food products. This study investigated the impact of incorporating mealworm (*Tenebrio molitor*) powder (MP) at 2%, 5%, and 10% levels on the nutritional, functional, and sensory properties of pasta. Proximate composition, mineral content, color parameters, cooking quality, antioxidant activity and sensory properties were evaluated. Starch digestibility fractions and predicted glycemic index (pGI) were calculated based on in vitro enzymatic hydrolysis. Results showed that 10% MP addition significantly increased protein (1.45-fold) and fat content (12-fold), enriched minerals (Fe, Zn, Mg, K), and improved antioxidant capacity (ABTS^+^·: 1.3-fold; DPPH·: 2.6-fold) and phenolic content (14.4-fold) compared to control. Color analysis revealed a decrease in lightness and an increase in redness, indicating darker tones with higher MP levels. This supplementation reduced rapidly digestible starch and pGI while increasing slowly digestible starch, suggesting benefits for glycemic control. Sensory evaluation revealed no significant differences (*p* > 0.05) among samples for appearance, color, taste, and overall impression, confirming good acceptability. Overall, MP fortification improved nutritional and functional properties without compromising sensory quality, supporting its application in developing high-protein, health-oriented foods.

## 1. Introduction

As the global population continues to grow and climate conditions change, along with other challenges, new food chain strategies are being developed to create sustainable food systems that are safe to consume and environmentally friendly. Insects are emerging as a promising addition to the European diet and have the potential to replace traditional protein sources partially. The increasing interest in entomophagy, or the practice of eating insects, is primarily due to their beneficial nutritional composition [1]. Another important aspect is that edible insects exhibit a very high feed-to-protein conversion efficiency, making them among the most efficient biomass-processing organisms [2]. Additionally, a significant advantage of edible insects is their ability to feed on waste or by-products from agricultural practices and the food industry. Insect farming requires significantly less water compared to traditional livestock farming. Specifically, it uses about 1.5 times less water than poultry farming, 2.5 times less than pig farming, and 5 times less than cattle farming to produce 1 kg of protein. Notably, some insects, such as *Tenebrio molitor*, do not require additional drinking water when raised at suitable humidity and fed a specific diet [3]. Additionally, edible insects produce significantly lower levels of greenhouse gases. The carbon dioxide equivalent (CO_2_-eq), which measures the contribution of different products to global warming, is approximately 12 kg CO_2_-eq for beef and only about 2 kg CO_2_-eq for edible insects [4]. Other advantages of insect biomass production include a faster growth rate, a short development cycle, high juvenile survival rates, a substantial egg-laying capacity, and an impressive potential for daily biomass growth [5].

Edible insects represent an innovative area of interest for enhancing nutrient supply, offering significant potential for global food security while providing an intriguing alternative to traditional meat products. Their incorporation into the food sector opens up a variety of applications. Firstly, insects are a valuable source of protein, especially for plant-based diets that rely on cereal proteins, which often lack essential amino acids such as lysine, threonine, and tryptophan. Furthermore, insects can serve as the foundation for developing innovative food products with higher protein content. Understanding the complete nutritional value of edible insects is essential for strategic planning and creating meals with optimized dietary profiles. This knowledge could lead to a shift in eating habits and an overall improvement in public health [6].

Research indicates that edible insects are a valuable source of protein, minerals, amino acids, and fatty acids, all of which are essential for human development. The nutritional value of edible insects can vary significantly across species due to their diversity. Additionally, factors such as the insects’ diet, habitat, and stage of metamorphosis can substantially influence their nutrient content [7].

In addition to their nutritional benefits, studies suggest that insects offer health benefits through bioactive compounds, such as phytosterols and polyphenols. Bioactive compounds are substances found in food that can influence physiological or cellular functions in both animals and humans. Furthermore, insect proteins, in addition to providing nutrients and essential amino acids, also offer bioactive peptides. In traditional medicine, particular species of edible insects are utilized to treat inflammatory diseases. Research has shown that consuming insects can help reduce intracellular production of reactive oxygen species and lipid peroxidation. Moreover, it can enhance the expression of Nrf2 and glutathione S-transferase proteins, which are crucial for the redox response to stress, especially following high glucose stimulation [8].

The consideration of insects as a viable alternative source of protein arises mainly from the increasing demand for energy and protein that future generations will encounter [9]. However, introducing insects into the food market faces significant challenges due to consumer acceptance. This reluctance is a substantial barrier to successfully launching food products that contain insects [10]. Consumer interest in eating edible insects may grow once they are processed, making them nearly indistinguishable from familiar foods like bread, biscuits, sauces, and soups [9].

Adding insect powder to traditional products can enhance their nutritional value and provide potential health benefits. Pasta is a widely consumed product used as a base for various enrichments, due to its popularity and the ease with which these additions can be prepared while maintaining its characteristics. Examples of ingredients used to enrich pasta include: agri-food by-products [11], leaves of edible wild plants [12], legume seeds [13], vegetables [14,15] and even fish [16]. Furthermore, in the case of pasta, consumer opinions and sensory analyses suggest that incorporating insects as an ingredient is beneficial for enrichment. Since pasta is typically consumed for its energy content, incorporating nutritious ingredients like insects could enhance its appeal as a convenient food [17]. Current research on the application of mealworm powder in cereal products primarily focuses on individual indicators, such as physicochemical [18,19] or antioxidant properties [20]. The study aimed to evaluate the nutraceutical properties of pasta enriched with mealworm powder (*Tenebrio molitor*) at various levels. This study is the first to systematically investigate the synergistic effects on nutritional value, starch digestion characteristics, predicted glycemic index, and antioxidant activity. Additionally, it analyzes the color, culinary properties, and sensory aspects, which serve as a foundation for developing a new product recipe. Overall, the study evaluates the physical, nutritional, bioactive, and sensory properties, providing valuable insights about the newly developed product that can be adapted for commercial production.

## 2. Results and Discussion

### 2.1. Pasta Cooking Quality

Cooking properties are key indicators of pasta quality, and recipe modifications, especially increases in protein content, can significantly influence its characteristics. The impact of substituting durum wheat semolina with mealworm powder on pasta cooking quality is presented in Table 1. As the amount of mealworm powder (MP) additive increased, noticeable changes were observed in the pasta’s technological parameters. The optimal cooking time increased from 7.5 min in the control sample to 9.05 min in the P-MP10 variant. The difference in cooking times between the supplemented pasta samples and the control sample may be due to higher protein and lower starch content. This combination results in a slower rate of water absorption, which in turn raises the optimal cooking times [21]. Some authors have reported that adding insect powder to pasta can result in longer optimal cooking times [18,21,22].

Additionally, increasing the MP content significantly reduced the cooking weight. While the control sample and P-MP2 did not show statistically significant differences, both P-MP5 and P-MP10 had notably lower values (*p* < 0.05). Cabuk and Yilmaz [21] had similar observations for pasta enriched with mealworm powder. Previous observations have shown that adding various high-protein flours affects the water-absorption capacity of food products. This effect mainly depends on the protein amino acid composition, the dietary fiber and starch content, and the amylose/amylopectin ratio of the flours [23].

At the same time, cooking losses decreased from 4.09% in the control sample to 3.10% in the P-MP10 variant, with all samples differing significantly from one another. Generally, the lower the cooking loss, the higher the quality of the pasta. The increased protein content in the pasta may provide a better structure for denatured proteins, allowing for more effective starch capture and reducing losses during cooking [23].

These results suggest that higher levels of MP enhance the product’s stability during cooking, leading to reduced losses, although they also result in less swelling and require longer cooking times.

### 2.2. Color Measurements

The color of pasta plays a significant role in the selection process, influencing consumer preferences and expectations. It is one of the sensory characteristics that is easiest and quickest to evaluate. Adding mealworm powder to pasta significantly alters its color. Even during cooking, the color differences in the pasta remain noticeable. The color of this pasta resembles that of whole semolina pasta, which is commonly perceived as healthier. This observation aligns with findings from other studies that have applied insect powder fortification [18,22].

**Figure 1:**
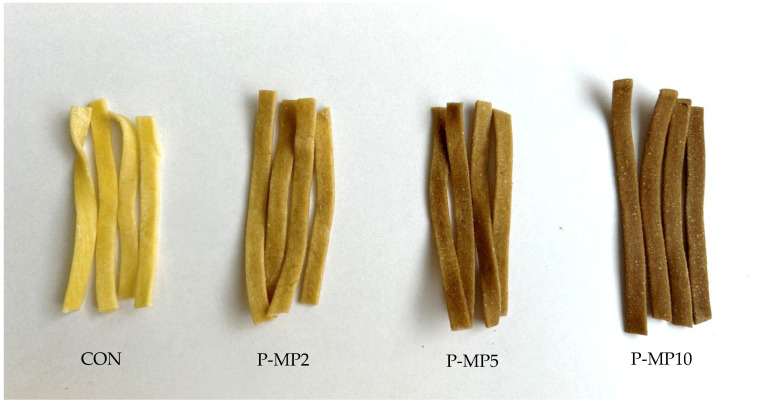
Control pasta (CON) and pasta with mealworm powder (P-MP2, P-MP5, P-MP10).

The effect of replacing durum wheat semolina with mealworm powder on color parameters of dry and cooked pasta is reported in Table 2. The highest lightness value was observed in the control sample (CON), as expected. As the amount of mealworm powder added increased, the lightness of the pasta decreased (*p* < 0.05), which is also visually noticeable (Figure 1). For the uncooked pasta samples, the lightness (L*) value decreased from 67.53 (control) to a range of 41.56 to 60.55. Similarly, for cooked pasta, the lightness values also reduced, ranging from 54.7 to 70.72, compared to the control value of 79.26. The observed changes are due to the presence of natural pigments in the powder. In the case of the mealworm, melanin is mainly responsible for the brownish color of the exoskeleton [24]. Additionally, while insects are slowly killed by freezing, browning reactions and other enzymatic changes occur [25].

The increase in lightness of cooked pasta relative to uncooked pasta was 17.37% for the control, 16.8% for P-MP2, 39.9% for P-MP5, and 31.6% for P-MP10. The increase in lightness may be attributed to losses that occur during the cooking process [26].

The a* parameter (redness) increased significantly—from 6.49 in CON to 12.19 in P-MP10, which indicates an intensification of red tones in the product. In turn, b* (yellowness) showed a downward trend—from 28.6 in the control sample to 23.2 in P-MP10, which means a reduction in the proportion of yellow color.

Calculating the total color difference (∆E) and the browning index (BI) is most effective for interpreting L*a*b* values. The ΔE index, which determines the total color difference relative to the control, reached its highest values in P-MP5 and P-MP10 (25.02–27.13), confirming significant visual changes. However, in all tests, the difference is noticed by an inexperienced observer because the value ΔE exceeds 2 [27]. The browning index (BI) increased more than 1.5-fold—from 60.84 in CON to 99.97 in P-MP10—which indicates an intensification of dark tones. Similar results were reported by other researchers who evaluated pasta containing insect powder. Cabuk and Yılmaz [21] reported that substituting wheat flour with mealworm powder in traditional Turkish egg pasta resulted in a darker color compared to pasta made solely with wheat flour. Similar conclusions were drawn by Pasini et al., who enriched pasta with mealworm protein [18], while Duda et al. enriched pasta with cricket powder [22]. They recorded only slightly greater differences than in our case. ∆E values for pasta supplemented with 5% *Acheta domesticus* powder were 27.18, while for 10% supplementation, the value was 31.17. Pasini et al. [18] compared the enrichment of pasta with 14% of mealworm and cricket protein. They found that cricket protein had a higher total color difference (25.5) compared to mealworm protein (13.2). Insect protein extracts yielded darker, redder pasta colors, with less pronounced yellowness, consistent with our findings. The authors attribute the darker color of the pasta to enzymatic browning reactions, which were confirmed by Yi et al. in insect protein fractions [28].

After cooking the pasta, the a* parameter increased with the addition of flour powder, although to a lesser extent than in the dry state (from 2.1 to 5.73). At the same time, b* decreased from 17.76 in CON to 14.43 in P-MP10, indicating a reduction in yellow tones after cooking. The color difference ΔE between the cooked samples and the control was evident, especially in P-MP10 (25.05), and the browning index (BI) increased from 26.79 in CON to 37.83 in P-MP10. A comparison of ΔE between uncooked and corresponding cooked pasta shows that the most significant color changes resulting from heat treatment occurred in P-MP5 (20.51).

The addition of mealworm powder results in a noticeable darkening of the product and a shift toward red tones, which is typical of dark-colored protein additives. Cooking the pasta reduces the contrast but maintains a similar color change trend. These changes may affect consumer acceptance, so the visual aspect of the enrichment used should be carefully considered in product design.

### 2.3. Nutritional Value

The analysis of the chemical composition of the tested pasta samples revealed significant differences among the various pasta variants (*p* < 0.05) (Table 3). The protein content increased, respectively, with the level of mealworm powder added, rising from 10.76 ± 0.08% in the control sample (CON) to 15.61 ± 0.12% in the pasta with 10% mealworm powder. A similar trend was observed for fat content, which increased from 0.26 ± 0.02% in the CON to 3.12 ± 0.05% in the 10% mealworm pasta (P-MP10), with each successive dose of mealworm powder inducing a significant increase (*p* < 0.05). The ash content also increases with the addition of mealworm powder, reaching its highest value in P-MP10 at 2.78 ± 0.03%. This indicates that the product was enriched with minerals. Conversely, the carbohydrate content decreased from 74.92 ± 0.50% in the control sample to 66.13 ± 0.47% in P-MP10 (*p* < 0.05). This decrease is due to partial replacement of the starch fraction with a high-protein component. Additionally, the energy value increased from 345 ± 1.5 kcal/100 g in the CON sample to 356 ± 0.97 kcal/100 g in P-MP10 (*p* < 0.05), primarily due to the increased fat content.

For comparison, pure mealworm powder (MP) was characterized by very high protein (58.6 ± 0.12%) and fat (20.96 ± 0.2%) content, low carbohydrate content (6.74 ± 0.32%), and the highest energy value (450 ± 1.22 kcal/100 g). Therefore, a change in the pasta’s nutritional value was expected. Insects, as high-protein components, are proposed as an enrichment for many food product groups, and numerous scientific studies confirm their positive impact on the nutritional value of muffins [29], shortcake biscuits [30], bread [31,32,33], ice cream [34], pâté [35] and bars [36,37].

The addition of mealworm powder significantly enhances the product’s nutritional value by increasing its protein and fat content, which may be beneficial for enriching foods with high-protein ingredients. Additionally, the observed reduction in carbohydrates, along with a moderate increase in energy, suggests a shift in the nutritional profile toward a more energy-dense, high-structural-component product.

### 2.4. Mineral Content

Adding mealworm powder to the recipe had a clear impact on the product’s mineral content (Table 4). As the MP content increased, most of the analyzed elements also increased. For iron, the value increased from 1.67 mg/100 g in the control sample to 2.08 mg/100 g in the P-MP10 sample. Due to the relatively low content of this element in MP, supplementing pasta with small amounts (2% and 5%) did not result in a significant increase in iron content in the tested samples (*p* < 0.05) [38]. Noteworthy is the almost twofold increase in zinc content—from 1.74 mg/100 g in the control sample to 3.13 mg/100 g in P-MP10. Zinc plays a crucial role in enzyme function and immunity, and its increase in the product is beneficial from a nutritional standpoint [39]. Magnesium and potassium also showed a significant increase—magnesium from 64.48 mg/100 g to 83.31 mg/100 g, and potassium from 90.15 mg/100 g to 140.80 mg/100 g, which improves the electrolyte profile of the product and may support the proper functioning of muscles and the nervous system [40].

It is worth noting copper, whose content has more than doubled (from 0.40 mg/100 g to 1.12 mg/100 g), and sodium, which has increased from 4.52 mg/100 g to 12.03 mg/100 g.

For comparison, mealworm powder was exceptionally rich in minerals—it contained as much as 1301.07 mg/100 g of potassium, 319.06 mg/100 g of magnesium, as well as very high amounts of zinc (22.51 mg/100 g) and sodium (212.71 mg/100 g). Such a high concentration of minerals confirms that MP is a valuable source of micro- and macroelements.

The addition of MP significantly enriches the product with minerals, particularly iron, zinc, magnesium, and potassium, thereby enhancing its functionality. Edible insects are a rich source of minerals, but the level of mineral accumulation varies depending on the element, the species, the diet, and the metamorphosis stage of the insect. In general, the microelement content is higher in larvae than in adults, which may explain why larvae are preferred as food. Among 67 summarized insect species from orders such as Diptera, Coleoptera, Hemiptera, Hymenoptera, Isoptera, Lepidoptera, and Orthoptera *T. molitor* larvae showed the highest concentrations of Ca, K, Mg, P, Na, Zn, Cu, and Se [41].

### 2.5. Antioxidant Properties

Chronic oxidative stress is recognized as an important pathogenic factor in many diseases, including atherosclerosis, type 2 diabetes, and neurodegenerative diseases. Reactive oxygen species (ROS) participate in the oxidative degradation of lipids, proteins, and nucleic acids, leading to cellular structural damage and, consequently, to apoptosis or necrosis. Oxidative stress can be reduced by including compounds with antioxidant properties in the diet, such as plant polyphenols (e.g., quercetin, catechins), carotenoids (e.g., β-carotene, lycopene), and bioactive peptides. These compounds neutralize free radicals, chelate transition metal ions, and inhibit lipid peroxidation, supporting the body’s oxidative homeostasis [42]. Insects are also a rich source of bioactive compounds. Incorporating mealworm powder into the product significantly enhances its antioxidant properties (Table 5). The free radical scavenging capacity, measured using the ABTS^+^· and DPPH· methods, increased with increasing mealworm powder (MP) content. We observed a strong positive correlation between the addition of insect powder and the results from the ABTS^+^· and DPPH· tests, yielding R = 0.99 (*p* = 0.000) for both. In the ABTS^+^· test, the values rose from 0.586 mM TE in the control sample to 0.771 mM TE in the P-MP10 variant. In the DPPH· test, the values increased from 0.487 mM TE to 1.269 mM TE. The highest values were observed for pure mealworm powder, with 1.352 mM TE for ABTS^+^· and 2.007 mM TE for DPPH·, confirming its strong antioxidant potential.

A similar trend was noted regarding total polyphenol content (mg GAE/100 g). The control sample had only 0.82 mg GAE/100 g, while P-MP10 reached 11.79 mg GAE/100 g, an increase of more than fourteen times. We observe a strong positive correlation between the addition of insect powder and TPC, with R = 0.99 (*p* = 0.000). In contrast, mealworm powder itself exhibited an extraordinarily high polyphenol content of 419.9 mg GAE/100 g, confirming that MP is a rich source of bioactive compounds. Additionally, strong positive correlations were observed between TPC and the ABTS^+^· and DPPH· tests, with R = 0.98 for both (*p* = 0.000).

The results obtained align with findings from other insect studies that enrich food products. For example, the antioxidant activity levels of the shortcake biscuits or muffins increase as the concentration of mealworm flour in the recipe increased [29,30]. Navarro del Hierro et al. [43] demonstrated the existence of a statistically significant positive correlation between total phenolic content (TPC) and antioxidant activity of mealworm extracts. Our prior research on muffins enhanced with mealworm and cricket powder also confirmed a strong correlation in this area [29]. It is essential to highlight the observation by Di Mattia et al. [44] that the in vivo effectiveness of antioxidant-rich foods depends significantly on their bioavailability. The high antioxidant content of foods is a crucial factor in evaluating the antioxidant potential of new foods. Consequently, assessing the antioxidant potential of insect-enriched products in vitro at high concentrations provides valuable insights for further research, ultimately confirming this activity in vivo.

Insects contain various bioactive components that contribute to their antioxidant properties. These components include low-molecular-weight antioxidants, such as ascorbate, glutathione, and α-tocopherol, as well as antioxidant enzymes, such as superoxide dismutase, catalase, ascorbate peroxidase, and glutathione peroxidase [45]. Edible insects are a source of bioactive compounds, which are typically derived from plants. These include phenolic compounds, terpenoids, steroids, glycosides, organic acids, carotenoids, and sulfur compounds. Insects accumulate these compounds based on their diet during the larval stage [46]. In the study of the insect species *Tenebrio molitor*, researchers identified phenolic compounds in the skin and secretions of the defense glands [47]. Moreover, insect digestion leads to proteolysis of their proteins, yielding low-molecular-weight peptides with bioactive properties, including the ability to neutralize reactive oxygen species (ROS). Studies indicate that protein hydrolysates and peptides derived from edible insects exhibit high antioxidant activity, exceeding that of hydrolysates from plant- or animal-derived proteins. The mechanism of action of these peptides includes, among others, the ability to chelate transition metal ions, inhibit lipid peroxidation and scavenge free radicals. Previous analyses have mainly focused on the protein fraction of insects, including the amino acid composition of peptides, which determines their antioxidant properties [48].

Overall, the addition of mealworm powder significantly enhances the product’s antioxidant properties, potentially increasing its health benefits. The observed increase in antioxidant activity correlates with higher polyphenol content, a key compound that neutralizes free radicals.

### 2.6. Rapidly and Slowly Digestible Starch Contents and Predicted Glycemic Index

RDS refers to rapidly digestible starch, which is readily available and has been shown to predict the glycemic response to cereal-based foods [49]. In contrast, slowly digestible starch (SDS) refers to slowly released starch; although it provides carbohydrate energy, it does not elicit a high glycemic response in individuals without glucose intolerance [50]. The addition of mealworm powder to the product significantly altered the starch digestibility profile (Table 6). The content of rapidly digestible starch (RDS) decreased as the amount of mealworm powder increased, dropping from 132.1 mg/g in the control sample to 96.08 mg/g in the PM10 sample. This reduction makes the product less prone to rapid digestion, potentially reducing sharp spikes in blood glucose levels after consumption.

Conversely, the content of slowly digestible starch (SDS) increased, rising from 105.8 mg/g in the control sample to 124.0 mg/g in the P-MP10 sample. This increase in SDS is advantageous for stabilizing blood glucose levels, as slowly digesting starch releases glucose more evenly over time. The changes in SDS and RDS content were significant after adding 2% MP; however, further substantial changes were observed only at a 10% enrichment level. Enriching the pasta with 5% MP did not produce any significant differences compared to the P-MP2 sample. This suggests that the difference between the control sample and P-MP2 is greater than that between P-MP2 and P-MP5 in terms of components that interact with starch, ultimately affecting its digestion.

**Figure 2:**
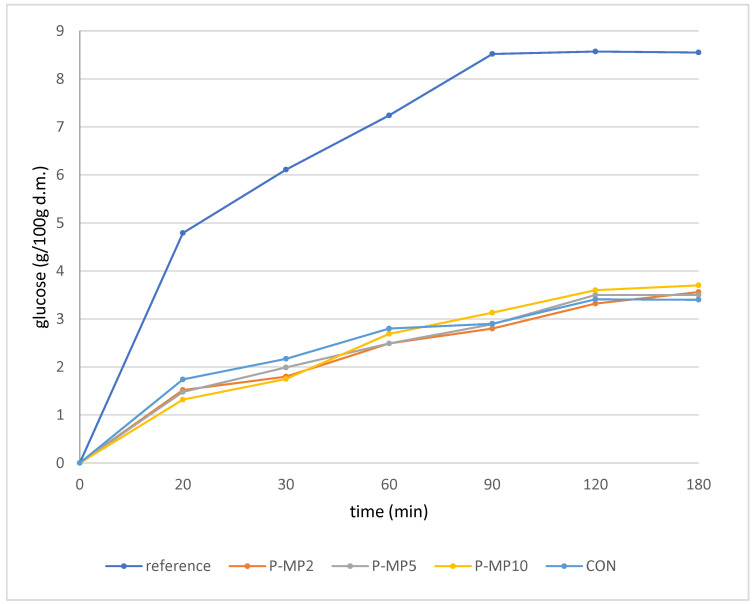
Glucose values from in vitro digestion of pasta samples and reference bread (g/100 g dry mass).

The glycemic index (GI) measures the increase in blood glucose levels after consuming carbohydrate-rich foods [51]. The glycemic index measures the glycemic response triggered by a specific amount of available carbohydrates in a test food compared to the response from the same amount of carbohydrates in a reference food. This assessment is carried out with the same individual consuming both foods [52]. The metabolic glycemic response to food products is estimated using the in vitro starch hydrolysis method. The predicted glycemic index (pGI) also decreased, falling from 52.53 in the control sample to 50.43 in the P-MP10 sample (Table 6). This indicates an improvement in the product’s nutritional quality regarding glycemic control. Although the differences are relatively small, the trend is clear: adding mealworm powder positively impacts the product’s metabolic properties. The amount of glucose released from starch during the in vitro digestion of pasta is shown in Figure 2. The patterns of the curves for all four pasta types were similar. As expected, the glucose values were significantly lower than those from the in vitro digestion of white breadcrumbs, which are presented in the same figure as a reference food. The differences in the predicted glycemic index were relatively small, contributing to the similarity in the glucose release curves. However, a slightly higher glucose level was observed during the initial stage of digestion in the control sample. In contrast, more glucose was released in the final stage of digestion for the P-MP10 sample.

Modulating postprandial blood glucose response through the consumption of low-glycemic index foods offers significant health benefits, underscoring the need for further research in this area. Several factors contribute to lowering the glycemic index, with the inclusion of additional food components being significant. The increased levels of protein, fat, and total polyphenols in the supplemented pasta may exert functional effects on carbohydrate digestion and absorption. Starch interacts with lipids and proteins via electrostatic and hydrophobic interactions, thereby enhancing its structural organization and reducing its digestibility. These interactions play a key role in determining starch digestibility and can occur under various conditions [53]. A significant increase in protein and fat content was observed in the supplemented pasta, which may be reflected in the predicted glycemic index results. Furthermore, phenolic compounds can inhibit amylolytic enzymes, thereby delaying glucose absorption and contributing to reduced postprandial glycemia [54]. The enriched pasta had a higher polyphenol content than the control sample, which may have influenced enzyme activity. Another potential mechanism, which can be observed here, involves the formation of viscous protein–fiber–starch networks that entrap starch granules, thereby limiting their accessibility to digestive enzymes [55]. Given the growing interest in low-glycemic-index products, these findings represent an important contribution to research on non-conventional protein sources, which, beyond their nutritional benefits, may also enhance the health-promoting properties of foods.

### 2.7. Consumer Acceptance Analysis

The senses of sight, taste, and smell are crucial in selecting food products. When designing new products enriched with edible insects, it is essential to conduct an organoleptic assessment with potential consumers. This assessment significantly influences consumer acceptance of the product. Analysis of the sensory evaluation results indicates that the addition of mealworm powder at the tested levels (2%, 5%, and 10%) did not result in statistically significant differences in any of the evaluated parameters (*p* > 0.05) (Table 7). All samples received high scores for appearance (4.55–4.78), suggesting that changes in color and structure did not adversely affect the product’s perceived appearance. Similarly, color was rated at a similar level (4.19–4.69), and the differences between samples were not significant, even though PM10 had the lowest value, which may be due to the intense darkening of color at the highest MP addition. The smell and taste also received high ratings (4.12–4.6 and 4.25–4.75, respectively), with no significant differences between samples, indicating good sensory acceptance across all variants. The overall impression remained high (4.3–4.55), confirming that the addition of MP does not compromise the product’s sensory quality.

A sensory analysis of durum wheat pasta enriched with *Acheta domesticus* powder demonstrated that this ingredient is suitable for pasta production. Untrained consumers rated the sensory attributes—such as color, taste, overall rating, and texture—higher than those of the control sample [22]. Similarly, Ho et al. [56] found that pasta made with *A. domesticus* powder was rated significantly higher for overall liking than the control group. Additionally, no significant differences were observed in the other tested attributes, including appearance, aroma, taste, texture, flavor, and aftertaste.

The lack of significant differences in sensory evaluation between studied samples indicates that the addition of MP, even at the highest level (P-MP10), is acceptable in terms of appearance, color, smell, taste and overall impression. These results suggest that the product can be enriched with edible insects without compromising its organoleptic characteristics.

### 2.8. Research Limitations and Future Prospects

This study has several limitations that should be acknowledged to ensure a more objective and practical interpretation of the findings. First, the long-term acceptance of insect-derived foods by consumers has not been fully evaluated. While short-term sensory assessments provide valuable insights, they do not capture long-term behavioral factors such as repeated purchase intent, cultural acceptance, or the effects of marketing and education. Consumer perceptions of insect-based foods may change gradually, so future studies should incorporate long-term evaluations to understand better how attitudes and willingness to buy may evolve over time.

An essential limitation of this study relates to the consumer analysis methodology. The sensory evaluation was carried out with a homogeneous group of young participants recruited from a university setting. This sample lacks demographic diversity across age, educational background, food habits, and cultural attitudes toward novel foods, limiting the generalizability of the findings. Younger, academically inclined individuals may be more receptive to food innovation. Additionally, informing participants about the presence of insect ingredients in the pasta may have influenced their responses—either positively, due to curiosity, or negatively, due to neophobia—thereby compromising the objectivity of the sensory assessment. Future research should involve a more diverse panel and include both blind and informed testing conditions to gather more reliable data on consumer acceptance.

The long-term stability of products made with insect powder requires further evaluation. The lipid composition of insect ingredients may make them more susceptible to oxidative changes, which can affect aroma, flavor, and safety of the products as they age. Research on processing methods and biorefinery techniques for *T. molitor* emphasizes the need for optimized stabilization and storage protocols to maintain quality during commercial distribution [57].

Estimating the large-scale production cost of *Tenebrio molitor* powder remains challenging. While mealworm powder is a promising protein source, and its production costs, in terms of feed and other resources, are lower than those of many animal proteins, the high initial investment required limits its immediate competitiveness in the market. Large-scale insect farming requires substantial financial investment in several areas, including automated breeding systems, genetic diversity control, intelligent climate control, and biosecure processing units [58].

Future research should focus on optimizing the addition levels of *Tenebrio molitor* powder to find the best balance between cost, nutritional benefits, and sensory quality. Identifying the most cost-effective concentration could help promote broader industry adoption. Furthermore, large-scale pilot studies that examine production efficiency, long-term consumer behavior, and extended shelf-life testing would provide valuable insights for the food industry. These studies could facilitate the integration of insect proteins into commercial food systems and aid in the development of stable, economically viable, and consumer-friendly products.

The results obtained highlight important conclusions; however, they do not fully capture the complexity of the issue under study. The limitations stemming from the methodology used and the type of supplementation indicate a need for further research. In future studies, it would be beneficial to expand the experiments to achieve a more accurate characterization of the observed processes. This approach will enhance the reliability and repeatability of the results, allowing for a more precise understanding of the mechanisms that underlie the analyzed phenomena and their significance within a broader scientific context.

## 3. Materials and Methods

### 3.1. Materials

Wheat flour semolina (protein 13%, fat 1.5%, carbohydrates 68%) (Antimo Caputo, Napoli, Italy) was purchased from the online shop. The mealworms *Tenebrio molitor* (Linnaeus, Coleoptera: Tenebrionidae) (larvae) were obtained from a commercial supplier from Poland (Bugstore, Kraków, Poland). Insects were kept at 25–28 °C and 50–60% humidity and fed wheat bran. Fresh fruits and vegetables were used as sources of water. Insects were collected after the 14th instar.

Trolox (6-hydroxy-2,5,7,8-tetramethylchroman-2-carboxylic acid), ABTS (2,20-azino-bis(3-ethylbenzothiazoline-6-sulfonic acid), DPPH (2,2-diphenyl-1-picrylhydrazyl), dinitrosalicylic acid, pepsin from porcine gastric mucosa (10080, 250 U/mg), pancreatin from porcine pancreas (P1750, 8 × USP specifications), amyloglucosidase (10115, 70 U/mg), and invertase (I4504, 300 U/mg) were purchased from Sigma-Aldrich Company, Ltd. (St Louis, MO, USA). The D-glucose oxidase/peroxidase assay kit (GOPOD format) was obtained from Megazyme International (Wicklow, Ireland). Standard solutions for minerals were obtained from Merck Millipore (Burlington, MA, USA). All other chemicals and reagents used were of analytical grade.

### 3.2. Production of Insect Powder

The insects were fasted for two days to empty their digestive tract, then they were frozen for 24 h at −18 °C and lyophilized (Delta 2–24 LSCplus, Christ, Osterode am Harz, Germany); process parameters: pressure 0.521 mbar, shelf temperature 20 °C, freeze-drying chamber temperature −65 °C, drying time 48 h. After lyophilization, insects were ground in a laboratory mill and passed through a 20-mesh sieve to obtain insect powder, which helps obtain a fraction optimal for most technological applications. The resulting powder was vacuum-packed and stored at −20 °C until further use.

### 3.3. Production of Pasta

The pasta was prepared in three variants with varying amounts of mealworm powder (MP) added to partially replace wheat flour at 2% (P-MP2), 5% (P-MP5), and 10% (P-MP10). The control pasta was made without insect powder (CON).

For each formulation, semolina pasta flour and distilled water were mixed in a domestic blender (Bosch, model MUM58365, Munich, Germany) at a flour-to-water ratio of 2.5:1 (*w*/*w*) for 5 min to achieve a homogeneous dough. The dough was then shaped and cut using a pasta machine (Hendi, model 224830, Robakowo, Poland). The pasta samples, which measured approximately 2 mm in thickness, 60 mm in length, and 5 mm in width, were dried in a laboratory dryer (Wamed, SUP-65, Warsaw, Poland) at 40 °C for 24 h. The final dried pasta had a residual moisture content of 12 g per 100 g.

Dried pasta (100 g) was cooked in 1000 mL of boiling water. After cooking, the pasta was drained and cooled to room temperature. The cooked pasta was then frozen at −20 °C, lyophilized using a laboratory freeze dryer and subsequently milled.

### 3.4. Pasta Cooking Quality

Dried pasta (100 g) was cooked in 1000 mL of boiling water. The optimum cooking time, cooking weight (CW), and cooking loss (CL) were determined according to the AACC method [59]. During cooking, the optimal cooking time was monitored until the white core of the pasta samples, visible in cross-section, disappeared. CW was calculated as the mass ratio of cooked and raw pasta (g/g). The drain water was collected, and CL was determined by testing the dry matter content in water after cooking [59].

### 3.5. Color Measurements

Color measurements were conducted using a colorimeter NH310 (3nh, Guangdong Threenh Technology Co.,Ltd., Guangzhou, China). The CIE L*a*b* scale recorded color differences in lightness (L*) and color (a*—redness, b*—yellowness). The total color difference (∆E) was calculated using the formula below:

(1) $$∆E=\;\sqrt{∆L^{2+}{∆a}^{2}+{∆b}^{2}},$$

where ΔL*, Δa*, and Δb* are differences in the L*, a*, and b* values between the reference sample and the test sample, respectively.

The browning index was calculated as follows:

(2) $$BI=\;\frac{100(x-0.31)}{0.17},$$

where x

(3) $$x=\frac{\left(a*+1.75L*\right)}{\left(5.645L*+a*-3.012b*\right)}$$

### 3.6. Nutritional Value

Moisture, ash, fat, and protein content were determined using standard analytical methods [60]. The carbohydrate content was calculated by difference, by subtracting the sum of ash, fat, protein, and moisture from 100%. The energy value was calculated using a conversion method [61]

### 3.7. Mineral Content

The concentrations of the most common minerals—Ca, Fe, K, Mg, Na, Cu and Zn—in pasta mineralizations were determined, following the method described by Jorhem and Engman [62]. 0.5 g of each cooked and lyophilized pasta sample was mixed with 4 mL of nitric acid (V). Then, the samples in closed vessels were mineralized in a microwave oven (Mars, Xpress, CEM Corporation, Matthews, NC, USA) (200 °C for 30 min). The minerals were quantitatively transferred to 25 mL volumetric flasks, which were filled up to the mark with deionized water. The concentration of mineral ions in the minerals was determined by flame atomic absorption spectrophotometry (FAAS, Solaar 939, Unicam, Ilminster, UK) at 422.7 nm for calcium, 285.2 nm for magnesium, 766.5 nm for potassium, 213.9 nm for zinc, 324.8 nm for copper, 589.0 nm for sodium, and 248.3 nm for iron using an air acetylene flame. The blanks were used as double blind samples in accordance with AOAC guidelines. Standard solutions for each element were used.

### 3.8. Antioxidant Properties

#### 3.8.1. Extraction of Bioactive Compounds

A 1 g portion of the lyophilized and finely ground pasta was extracted on a laboratory shaker for 120 min using 10 mL of a 4:1 ethanol–water (*v*/*v*) solution. The mixture was subsequently centrifuged at 3000 g for 10 min, and the resulting supernatant was stored at −20 °C until further analysis [63].

#### 3.8.2. DPPH (2,2-Difenylo-1-pikrylohydrazyl) Radical Scavenging Activity

The DPPH· scavenging activity was assessed using a modified version of the method developed by Brand-Williams et al. [64]. A 0.1 mL aliquot of the sample was combined with 2.9 mL of a 6 µM DPPH· working solution prepared in 75% methanol. After a 30 min reaction period, the absorbance was recorded at 515 nm. The scavenging activity (%) was calculated according to the following equation:

Scavenging activity (%) = [1 − (A sample/A control)] × 100,(4)

where A sample is the absorbance of the of sample–DPPH· mixture; A control is the absorbance of the DPPH· control solution.

The antioxidant capacity was expressed as Trolox Equivalent Antioxidant Activity (TEAC) and reported as mM Trolox per gram of sample.

#### 3.8.3. ABTS 2,2′-Azino-bis(3-ethylbenzothiazoline-6-sulfonic Acid) Radical Scavenging Activity

The ABTS^+^· radical scavenging activity was evaluated using a modified method described by Re et al. [65]. A 0.1 mL aliquot of the sample was mixed with 2.9 mL of the ABTS^+^· working solution, and after a 30 min reaction period, the absorbance was recorded at 734 nm. The scavenging activity (%) was calculated according to the following equation:

Scavenging activity (%) = [1 − (A sample/A control)] × 100,(5)

where A sample is the absorbance of the sample–ABTS^+^· mixture; A control is the absorbance of the ABTS^+^· control solution.

The antioxidant capacity was expressed as Trolox Equivalent Antioxidant Activity (TEAC) and reported as mM Trolox per gram of sample.

#### 3.8.4. Total Phenolic Content

The total phenolic content (TPC) of the samples was determined using the Folin–Ciocalteu spectrophotometric method, as described by Singleton and Rossi [66]. An aliquot of the extract (0.04 mL) was combined with 3.16 mL of distilled water and 0.2 mL of the Folin–Ciocalteu reagent. After thorough vortex agitation, 0.6 mL of saturated sodium carbonate solution was introduction into the mixture. The reaction mixture was subsequently incubated at 40 °C for 30 min. Absorbance was measured at 725 nm against a blank. The total phenolic content was calculated and expressed as milligrams of gallic acid equivalents (GAE) per gram of sample.

### 3.9. In Vitro Digestibility of Starch

The in vitro digestibility of starch was evaluated according to a slightly modified procedure described by Monro et al. [67]. In brief, cooked pasta (1 g) was combined with 30 mL of water and 0.8 mL of 1 M HCl in order to adjust the pH to 2.5. Gastric digestion was then initiated by the addition of 1 mL of a 10% pepsin solution prepared in 0.05 M HCl. The mixture was maintained at 37 °C for 30 min under continuous stirring at 130 rpm.

To simulate the small intestinal phase, 2 mL of 1 M NaHCO_3_ and 5 mL of 0.1 M phosphate buffer (pH 6) were subsequently added, followed by the introduction of 4.6 mg of amyloglucosidase and 5 mL of 2.5% pancreatin prepared in 0.1 M phosphate buffer (pH 6). The volume of the resulting hydrolysates was then brought to 55 mL with distilled water.

Aliquots of 1.0 mL were withdrawn at 0, 20, 30, 60, 90, 120, and 180 min from the onset of amylolysis. Each aliquot was immediately combined with 4 mL of absolute ethanol to inactivate enzyme activity. The samples were then centrifuged, and the supernatants obtained were used to determine the rapidly and slowly digested starch contents and predicted glycemic index.

### 3.10. Rapidly and Slowly Digested Starch Contents

The levels of rapidly digestible starch (RDS) and slowly digestible starch (SDS) were quantified by measuring the reducing sugars released during the in vitro digestion of pasta, following a modified method described by Soong et al. [68]. The released sugars, measured as monosaccharides, were analysed using the dinitrosalicylic acid (DNS) colorimetric method.

The concentration of released sugars was reported as milligrams of glucose per gram of sample. RDS was defined as the amount of reducing sugars released after 20 min of intestinal digestion, whereas SDS was calculated as the difference between the reducing sugars measured after 120 min and the RDS value.

To ensure complete depolymerization into monosaccharides, 50 µL of the supernatant was combined with 0.25 mL of acetate buffer containing 0.4% invertase and 1% amyloglucosidase, followed by incubation at room temperature for 30 min. Subsequently, 0.75 mL of the DNS mixture (0.5 mg/mL glucose, 4 M NaOH, and DNS reagent in a 1:1:5 ratio) was added, and the samples were heated at 95–100 °C for 15 min. After cooling, the samples were diluted with 4 mL of distilled water, and absorbance was recorded at 530 nm against a blank.

### 3.11. Glycemic Index In Vitro

The in vitro glycemic index (GI) of pasta was determined using the method described by Reis and Abu-Ghannam [69]. Cooked pasta samples were subjected to enzymatic digestion as described above (3.8).

The glucose content of the samples was quantified using the glucose oxidase/peroxidase (GOPOD) assay and plotted as a function of time. Areas under the hydrolysis curves (AUC) were subsequently calculated, and hydrolysis index (HI) values were expressed as percentages by comparing the AUC of each sample’s AUC to that of the reference food (white bread). The HI values were normalised according to the total available carbohydrate content of both the sample and the reference. The glycemic index was then calculated using the equation proposed by Goñi, Garcia-Alonso, and Saura-Calixto [70].

GI (%) = 39.71 + 0.549 × HI,(6)

### 3.12. Consumer Acceptance Analysis

The semi-consumer evaluation was carried out with a panel of 70 participants aged 19–36 years, randomly selected from students of the University of Life Sciences in Lublin (Poland). Prior to participation, all individuals received written Information for Study Participants and signed an Informed Consent Form. Consumers were informed of the presence of insects in the evaluated pasta and of the possibility of allergic reactions for those with shellfish and dust mite allergies. The color (uniformity, intensity, product conformity, color appeal), aroma (intensity, purity, compliance with the nature of the product, complexity and appeal), appearance (shape and integrity, surface texture and surface smoothness, visual consistency, visual appeal), taste (intensity, harmony and balance, purity of taste, aftertaste, compliance with product type), and overall acceptability of the pasta (overall impression of enjoyment of consumption, willingness to try the product again, balance of all sensory characteristics as a whole, compliance with consumer expectations) were assessed on a hedonic scale of 1 to 5 (5—excellent, 1—extremely unsatisfactory). All evaluation conditions—including the laboratory environment, sample portion size, coding system, and serving plates—were maintained consistently throughout the study to ensure the comparability and reliability of the results.

### 3.13. Statistical Analysis

All assays were conducted in triplicate, and the results are presented as means ± SEM (standard error of the mean). Statistical analyses were performed using Statistica software (version 13.0, StatSoft, Kraków, Poland). Differences between means were evaluated using one-way ANOVA, followed by Tukey’s Honestly Significant Difference (HSD) post hoc test, with a significance level set at *p* < 0.05. The insect flour addition and TPC were compared with the values obtained for antioxidant capacity (ABTS^+^· and DPPH·) using Pearson’s correlation test.

## 4. Conclusions

Edible insects are gaining popularity for their ability to enhance traditional foods with nutrients. However, their role as food components extends beyond nutritional value, as they also contribute to food’s health-promoting properties. This study evaluated the potential of edible insects to enhance the nutritional value and health-promoting properties of pasta. The addition of 2%, 5%, and 10% mealworm powder significantly impacted the pasta’s nutritional, functional, and health-promoting properties. An increase in MP content led to notable rises in both protein and fat levels, as well as enhancements in the mineral profile, particularly for iron (Fe), zinc (Zn), magnesium (Mg), and potassium (K). Conversely, carbohydrate content decreased, leading to a moderate increase in the product’s overall energy value. Color analysis indicated a darkening effect (a decrease in L* and an increase in a*), yet sensory evaluations found no significant differences between the samples (*p* > 0.05), confirming that all variants were well accepted.

The addition of MP significantly enhanced antioxidant activity, as measured by ABTS^+^· and DPPH· assays, and increased polyphenol content, highlighting the product’s potential health benefits. Additionally, the observed reduction in the rapidly digestible starch (RDS) fraction and the decrease in the predicted glycemic index (pGI), along with a corresponding rise in slowly digestible starch (SDS), suggest a positive impact on glycemic control.

In summary, adding mealworm powder enriches the product’s nutritional and functional value while maintaining its sensory characteristics, making this approach promising for developing high-protein foods with improved health benefits.

## Figures and Tables

**Table 1:** Pasta cooking quality.

	CON	P-MP2	P-MP5	P-MP10
Optimal cooking time (min)	7.5 ± 0.08 ^b^	8.9 ± 0.07 ^a^	9.0 ± 0.06 ^a^	9.05 ± 0.1 ^a^
Cooking weight (g/g)	2.25 ± 0.11 ^a^	2.05 ± 0.07 ^a^	1.78 ± 0.12 ^b^	1.69 ± 0.06 ^b^
Cooking loss (%)	4.09 ± 0.14 ^a^	3.84 ± 0.08 ^a^	3.49 ± 0.7 ^b^	3.1 ± 0.08 ^c^

^1^ Values with different letters within the same row are significantly different (*p* ≤ 0.05).

**Table 2:** Color parameters (L*, a*, b*), color difference (∆E) and browning index (BI) of dry and cooked pasta.

	CON	P-MP2	P-MP5	P-MP10
dry				
L*	67.53 ± 0.51 ^aB^	60.55 ± 0.67 ^bB^	43.44 ± 0.37 ^cB^	41.56 ± 0.64 ^cB^
a*	6.49 ± 0.44 ^cA^	8.39 ± 0.48 ^bA^	11.69 ± 0.7 ^aA^	12.19 ± 0.08 ^aA^
b*	28.6 ± 0.5 ^aA^	27.26 ± 0.62 ^aA^	24.29 ± 0.39 ^bA^	23.2 ± 0.38 ^bA^
∆E	-	7.36 ± 1.49 ^b^	25.02 ± 2.54 ^a^	27.13 ± 2.1 ^a^
BI	60.84 ± 2.26 ^c^	68.56 ± 2.71 ^b^	98.45 ± 2.48 ^a^	99.97 ± 1.5 ^a^
cooked				
L*	79.26 ± 0.52 ^aA^	70.72 ± 0.48 ^bA^	60.78 ± 0.5 ^cA^	54.7 ± 0.46 ^dA^
a*	2.1 ± 0.14 ^cB^	4.57 ± 0.21 ^bB^	5.33 ± 0.2 ^aB^	5.73 ± 0.05 ^aB^
b*	17.76 ± 0.22 ^aB^	17.66 ± 0.16 ^aB^	15.39 ± 0.4 ^bB^	14.43 ± 0.3 ^bB^
∆E	-	8.95 ± 0.84 ^c^	18.91 ± 1.41 ^b^	25.05 ± 1.63 ^a^
BI	26.79 ± 2.25 ^b^	34.85 ± 0.72 ^a^	35.15 ± 1.16 ^a^	37.83 ± 0.97 ^a^
∆E dry—cooked	16.57 ± 0.98 ^b^	13.85 ± 0.85 ^c^	20.51 ± 1.05 ^a^	17.06 ± 0.94 ^b^

^1^ Values with different lowercase letters within the same row are significantly different (*p* ≤ 0.05). Values with different uppercase letters within the same column related to the same parameter are significantly different (*p* ≤ 0.05).

**Table 3:** Nutritional value of dry pasta (%).

	Protein	Fat	Ash	Carbohydrates	Moisture	Energy kcal/100 g
CON	10.76 ± 0.08 ^d^	0.26 ± 0.02 ^d^	2.02 ± 0.03 ^d^	74.92 ± 0.5 ^a^	12.05 ± 0.06 ^b^	345 ± 1.5 ^c^
P-MP2	13.36 ± 0.1 ^c^	0.76 ± 0.03 ^c^	2.26 ± 0.02 ^c^	71.79 ± 0.44 ^b^	11.83 ± 0.11 ^b^	347 ± 1.29 ^bc^
P-MP5	14.26 ± 0.11 ^b^	1.84 ± 0.03 ^b^	2.47 ± 0.06 ^b^	68.91 ± 0.51 ^c^	12.52 ± 0.17 ^a^	351 ± 2.83 ^b^
P-MP10	15.61 ± 0.12 ^a^	3.12 ± 0.05 ^a^	2.78 ± 0.03 ^a^	66.13 ± 0.47 ^d^	12.36 ± 0.08 ^a^	356 ± 0.97 ^a^
MP	58.6 ± 0.12	20.96 ± 0.2	4.56 ± 0.05	6.74 ± 0.32	9.14 ± 0.2	450 ± 1.22

^1^ Values with different letters within the same column are significantly different (*p* ≤ 0.05).

**Table 4:** Mineral composition of cooked pasta (mg/100 g d.w.).

	Minerals (mg/100 g d.w.)						
	Fe	Ca	Zn	Mg	K	Cu	Na
CON	1.67 ± 0.08 ^b^	76.02 ± 3.80 ^a^	1.74 ± 0.09 ^c^	64.48 ± 3.12 ^c^	90.15 ± 4.12 ^c^	0.40 ± 0.02 ^c^	4.52 ± 0.23 ^d^
P-MP2	1.86 ± 0.09 ^ab^	83.82 ± 4.2 ^a^	1.78 ± 0.09 ^c^	69.68 ± 3.5 ^bc^	124.5 ± 5.20 ^b^	0.46 ± 0.02 ^c^	5.75 ± 0.3 ^c^
P-MP5	1.89 ± 0.09 ^ab^	84.69 ± 4.23 ^a^	2.30 ± 0.12 ^b^	78.33 ± 3.92 ^ab^	126.79 ± 6.11 ^ab^	0.59 ± 0.03 ^b^	8.18 ± 0.4 ^b^
P-MP10	2.08 ± 0.10 ^a^	82.31 ± 5.1 ^a^	3.13 ± 0.11 ^a^	83.31 ± 4.17 ^a^	140.80 ± 7.04 ^a^	1.12 ± 0.06 ^a^	12.03 ± 0.6 ^a^
MP	6.37 ± 0.2	65.32 ± 3.3	22.51 ± 0.2	319.06 ± 6.95	1301.07 ± 10.05	2.16 ± 0.08	212.71 ± 3.64

^1^ Values with different letters within the same column are significantly different (*p* ≤ 0.05).

**Table 5:** Antioxidant properties of cooked pasta.

	ABTS^+^· (mM TE)	DPPH· (mM TE)	TPC (mg GAE/100 g)
CON	0.59 ± 0.06 ^d^	0.49 ± 0.08 ^c^	0.82 ± 0.05 ^d^
P-MP2	0.63 ± 0.06 ^c^	0.67 ± 0.01 ^c^	4.55 ± 0.2 ^c^
P-MP5	0.69 ± 0.06 ^b^	0.88 ± 0.04 ^b^	7.19 ± 0.23 ^b^
P-MP10	0.77 ± 0.05 ^a^	1.27 ± 0.03 ^a^	11.79 ± 0.41 ^a^
MP	1.35 ± 0.08	2.01 ± 0.09	419.9 ± 2.92

^1^ Values with different letters within the same column are significantly different (*p* ≤ 0.05).

**Table 6:** Starch digestibility and predicted glycemic index.

	RDS (mg/g)	SDS (mg/g)	pGI
CON	132.1 ± 0.31 ^a^	105.8 ± 1.6 ^c^	52.53 ± 0.32 ^a^
P-MP2	103.0 ± 1.58 ^b^	112.95 ± 1.31 ^b^	51.66 ± 0.24 ^b^
P-MP5	102.6 ± 0.76 ^b^	116.92 ± 2.5 ^b^	51.47 ± 0.31 ^b^
P-MP10	96.08 ± 0.23 ^c^	124.0 ± 1.27 ^a^	50.43 ± 0.26 ^c^

^1^ Values with different letters within the same column are significantly different (*p* ≤ 0.05).

**Table 7:** Consumer acceptance analysis.

	CON	P-MP2	P-MP5	P-MP10
appearance	4.75 ± 0.51 ^a^	4.55 ± 0.67 ^a^	4.78 ± 0.37 ^a^	4.56 ± 0.64 ^a^
color	4.49 ± 0.44 ^a^	4.39 ± 0.48 ^a^	4.69 ± 0.7 ^a^	4.19 ± 0.48 ^a^
smell	4.6 ± 0.5 ^a^	4.26 ± 0.62 ^a^	4.2 ± 0.39 ^a^	4.12 ± 0.38 ^a^
taste	4.65 ± 0.55 ^a^	4.75 ± 0.51 ^a^	4.25 ± 0.44 ^a^	4.26 ± 0.35 ^a^
overall impression	4.55 ± 0.44 ^a^	4.52 ± 0.52 ^a^	4.3 ± 0.48 ^a^	4.37 ± 0.41 ^a^

^1^ Values with different letters within the same row are significantly different (*p* ≤ 0.05).

## Data Availability

The original contributions presented in the study are included in the article; further inquiries can be directed to the corresponding author.

## Abbreviations

The following abbreviations are used in this manuscript:

∆E	color difference
ABTS^+^·	2,20-azino-bis(3-ethylbenzothiazoline-6-sulfonic acid) radical
BI	browning index
CON	control pasta
DPPH·	2,2-diphenyl-1-picrylhydrazyl radical
GOPOD	glucose oxidase/peroxidase
MP	mealworm powder
pGI	predicted glycemic index
P-MP2	pasta with 2% mealworm powder
P-MP5	pasta with 5% mealworm powder
P-MP10	pasta with 10% mealworm powder
RDS	rapidly digestible starch
SDS	slowly digestible starch
TEAC	Trolox Equivalent Antioxidant Activity
